# Prognostic value of different N1 lymph node zones in pN1M0 non-small cell lung cancer: a systematic review and meta-analysis

**DOI:** 10.1038/s41598-021-01136-2

**Published:** 2021-11-03

**Authors:** Fengling Hu, Chutong Lin, Hongling Chu, Peng Ren, Jingdi Wang, Shaohua Ma

**Affiliations:** 1 Department of Thoracic Surgery, Peking University Third Hospital, 49 North Garden Rd., Haidian District, Beijing, 100191 People’s Republic of China; 2 Research Center of Clinical Epidemiology, Peking University Third Hospital, 49 North Garden Rd., Haidian District, Beijing, People’s Republic of China

**Keywords:** Cancer, Lung cancer

## Abstract

The IASLC lymph node map grouped the lymph node stations into “zones” for prognostic analyses. In the N1 lymph nodes group, N1 nodes are divided into the Hilar/Interlobar zone (N1h) and Peripheral zone (N1p). There is no consensus on the different prognostic values of N1 lymph nodes in N1h and N1p. Therefore, we conducted a systematic review and meta-analysis to assess the survival difference between N1h and N1p in patients of pN1M0 NSCLC. Medline, the Cochrane Library, Embase, and the Web of science were systematically searched to identify relevant studies published up to April 4th, 2020. A retrospective and prospective cohort study comparing N1h versus N1p to the pN1M0 NSCLC was included. Hazard ratios (HRs) for OS were aggregated according to a fixed or random-effect model. Ten publications for 1946 patients of pN1M0 NSCLC were included for the meta-analysis.The 5-year OS was lower for patients with N1h (HR: 1.67, 95% CI 1.44–1.94; *P* < 0.001). The pooled 5-year OS in N1h and N1p were 40% and 56%, respectively. The patients in pN1M0 NSCLC have different survival according to different N1 lymph node zones involvement: patients with N1p metastasis have a better prognosis than those with N1h metastasis.

## Introduction

Lung cancer is the leading cause of cancer-related deaths worldwide. Non-small cell lung cancer (NSCLC) accounts for more than 85% of all lung cancer pathologic types, and adenocarcinoma is the primary pathological type of non-small cell lung cancer^1^.

TNM classification for lung cancer was introduced in 1973 and has been widely used since the 5th edition in 1997^2^. In TNM classification, N descriptors are one of the most important prognostic indicators, but relative to the frequent revision of the T descriptors, N descriptors changes have been rarely: In 1985, N1 was described as “ipsilateral peribronchial, interlobar, or hilar node involvement” and N1 descriptors had not changed until the eighth edition of TNM classification was put forward. In the 8th edition of the TNM classification, the N1 was divided into N1a and N1b to distinguish between the single station and multiple stations^3^.

In the current TNM NSCLC staging system, the N descriptors are determined by the metastatic lymph node stations using the International Association for the Study of Lung Cancer (IASLC) lymph node map, which was developed to reconcile differences among the Naruke map and Mountain-Dresler modification of the ATS map (MD-ATS) in 2009^4^.

In the IASLC lymph node map, IASLC grouped lymph node stations into “zones” to inquiry about different potential prognoses. In the N1 lymph nodes group, N1 nodes are divided into Hilar/Interlobar zone (#10–11) and Peripheral zone (#12–14)^4^. Some authors have recently proposed a different prognosis for N1 lymph node involvement in the Hilar/Interlobar zone and Peripheral zone^5–8^. However, there is no consensus on the different prognostic values of N1 lymph nodes in the Hilar/Interlobar and Peripheral zones. For the sake of brevity, we stratified pN1 patients into two categories according to the zones of metastatic LNs as follows: N1h, pN1 status with lymph node involvement in the Hilar/Interlobar zone (whether Peripheral zone involves); N1h, pN1 status with lymph node involvement in the peripheral zone. Therefore, we conducted a systematic review and meta-analysis to try to answer the following questions:

Is there a survival difference between N1h and N1p in patients of pN1M0 NSCLC? Do we need more detailed N1 classifications to distinguish heterogeneity among pN1M0 patients?

## Methods

### Search strategy

Before conducting the meta-analysis, we registered with the International Prospective Register of Systematic Reviews and submitted the system review protocol (PROSPERO CRD42020178874). In systematic reviews, we strictly follow the Preferred Reporting Items for Systematic Reviews and Meta-Analyses guidelines^9^. To identify eligible studies of patients with N1 lymph nodes involved, in this study, we searched Medline (through PubMed), the Cochrane Library, Embase, and the Web of Science from inception January 1st, 2000 to April 4th, 2020. We used a combination of MeSH terms and free-text words for “NSCLC”, “lymph node”, and “N1”. The search queries were built by the advice of the Hedges Project of McMaster PLUS Projects^10^ (Supplemental Table).

### Inclusion criteria and exclusion criteria

This systematic review incorporated studies that meet the following inclusion criteria: (1) participants: pN1M0 NSCLC patients undergoing pulmonary resection with lymph node dissection; (2) research data: survival data between N1p and N1h patients; (3) primary outcomes: the 5-year overall survival (OS) and disease-free survival (DFS); (4) research categories: prospective or retrospective cohort study; Exclusion criteria were: (1) No N1p and N1h grouping information (2) necessary survival data were lacking for statistics, including hazard ratio (HR), 95% confidence interval (CI)or Kaplan–Meier curve; (3) reviews, case reports, conference abstracts, comments, and letters; (4) duplicate publications or data sources.

### Data extraction

Two authors (FLH and CTL) independently searched the databases and checked the retrieved literature titles and abstracts. Perhaps relevant articles would be included in our selection of full-text reading. When there was any disagreement, the disagreement would be resolved with the help of the third author (SHM). If the disagreement cannot be resolved, it would be resolved by consultation with the statistical expert (HLC) and group discussions. In highly relevant articles, we will carefully read their citations to detect other studies we interest in.

Study characteristics (first author, year of publication, country, research types, number of patients, stage of primary T, surgical method, additional treatment, the principle of lymph node classification) and outcome (5-year OS, HR for DFS and OS) data were extracted from the included articles. We discussed any discrepancies and reached a consensus.

When there are both univariate and multivariate analyses results, results from the multivariate analyses were preferred. When HR for OS or PFS was not available, it would be estimated from the Kaplan–Meier curves using the approach described by Tierney et al.^11^ using digitized software and the spreadsheet attached to repeat the calculation twice independently to ensure the consistency of the results. Two authors (FLH and CTL) used the Newcastle–Ottawa scale to assess the risk of bias independently^12^.

### Data analysis

After data extraction, we found that few studies reported DFS, so we used 5-year OS as the primary outcome to evaluate the prognostic difference between N1h and N1p. According to a fixed or random-effect model, hazard ratios for OS, comparing the N1p group versus N1h group, were aggregated in a final meta-analysis. The 5-year OS was pooled using weight calculated from the OS and standard error (SE). The heterogeneity was assessed using *I*^2^ statistics. *P* values < 0.05 or *I*^2^ values > 50% would be considered indicative of heterogeneity. An appropriate statistical method would be selected based on the heterogeneity degree to summarize the rates and corresponding 95% confidence intervals (CIs). In all statistical analyses, *P* values are calculated using two-tailed tests. Funnel plot analysis and Egger’s tests were used to evaluate publication bias. All analyses were performed with Stata/SE 14.0 and the meta-packages.

## Results

### Study selection

**Figure 1:**
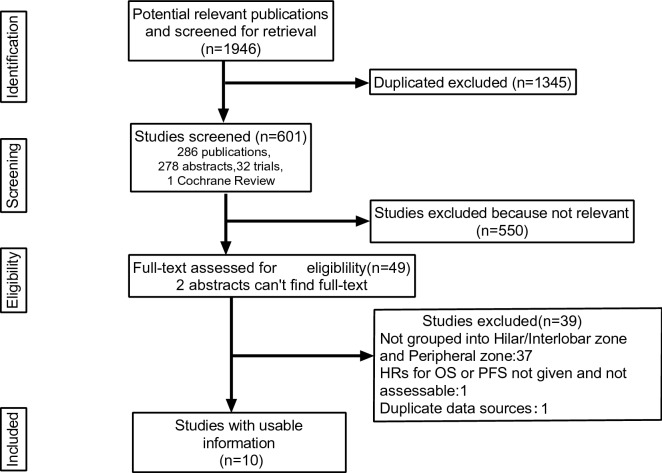
Literature search flow diagram.

A total of 1946 results were identified in the systematic research. After checking the author, title, and abstract for the first time, 1345 duplicate documents were excluded. In the second sifting, 550 studies were excluded based on the titles and abstracts. In the remaining 51 articles, two abstracts cannot find the full text. We carefully reviewed the remaining 49 articles according to the above inclusion and exclusion criteria. Among the remaining studies, 37 studies did not group into N1h, and N1p were excluded. One study was further excluded because the HR for DFS and OS was unreported and unreachable. Two other studies used patient information from the same database, so we excluded a study with fewer patients. Finally, a total of 10 studies^6,13–21^ were identified in the meta-analysis. The PRISMA statement of the selection flow diagram is shown in Fig. 1.

### Characteristics of included studies

Among the ten studies, a total of 1946 pN1M0 patients (47.7% in N1h and 52.3% in N1p) accepted lung resection (Table 1), almost all patients were accepted anatomical lung resection and systematic nodal dissection. Most patients (88.6%) were in the stage of primary T1–2 versus 11.4% who were in pT3–4. In four publications, the additional treatment was not reported. In the rest of the six studies, 488 patients (43.5%) received additional treatments, and the remaining 635 patients (56.5%) did not receive additional therapy. All patients receiving additional therapy are concentrated in two studies, three studies mainly focused on patients who did not receive additional treatment, and one study remained balanced in additional therapy. N1 lymph nodes were defined according to the lymph node map published by the International Association for the Study of Lung Cancer (IASLC) in three studies, and there are other four studies according to the Naruke map and two studies according to the Mountain-Dresler modification of the American Thoracic Society (MD-ATS) map. The quality of cohort studies was high (median 7.5).

**Table 1:** Characteristics of the Studies Included in the Meta-Analysis.

#	First author	Year	No. of patients	No. of patients (N1h/N1p)	Stage of primary T% T1–2/T3–4	Additional treatment % yes/no	Lymph nodes defined according	HR source	HR [95%CI]	Quality score
1^13^	Maeda, H	2009	319	148/171	319/0	24/319	Naruke map	Available	1.41 [1.00–1.98]	6
2^6^	Liu, C. Y	2012	163	77/86	163/0	163/0	IASLC	Available	2.595 [1.507–4.469]	8
3^6^	Moon, Y	2018	32	9/23	28/4	0/32	IASCL	Available	6.848 [1.458–32.161]	9
4^15^	Shimada, Y	2009	202	78/124	171/31	–	Naruke map	Estimated	1.36 [0.80–2.10]	7
5^16^	Demir, A	2009	468	219/249	330/138	–	MD-ATS	Available	1.4 [0.8–2.1]	8
6^17^	Li, Z. M	2013	184	74/110	184/0	206/0	IASLC	Estimated	1.85 [1.24–2.76]	8
7^18^	Gonfiotti, A	2008	157	103/54	130/26	15/157	MD-ATS	Available	1.43 [0.94–2.19]	7
8^19^	Okada, M	2005	127	73/54	–	–	Naruke map	Estimated	1.68[1.00–2.82]	6
9^20^	Haney, J. C	2014	230	122/108	230/0	102/127	IASLC	Available	2.02 [1.40–2.91]	8
10^21^	Citak, N	2015	64	25/39	–	–	IASLC	Estimated	1.56 [0.76–3.19]	6

### Meta-analysis of OS

**Figure 2:**
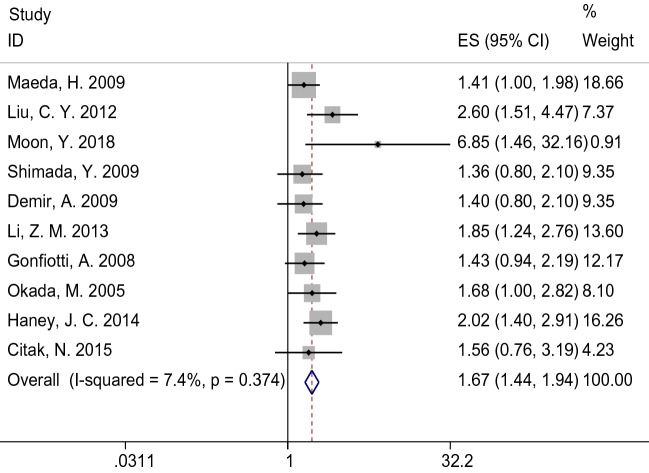
Forest plot of hazard ratios for overall survival of patients diagnosed with pN1 NSCLC and involved with N1h versus N1p. *CI* confidence interval, *HR* hazard ratio.

In 6 studies, HRs for OS were directly given, and the HRs in the remaining four studies were assessed using Kaplan–Meier curves. Observed HRs ranged from 1.4 to 6.848. Most of the studies were medium in size except for a small sample study. The heterogeneity factor, *I*^2^ was 7.6% (*P* = 0.374), which corresponds to low heterogeneity. So, the fixed-effects model yielded a pooled HR of 1.67 (95% CI 1.44–1.94, *P* < 0.01), suggesting the patients with N1p involvement tend to show better survival than patients with N1h involvement (Fig. 2). The sensitivity analysis results showed that the pooled results were robust (Supplementary Fig. S1). There was no significant publication bias among the studies for 10 study groups (Egger’s test: *P* value = 0.149). The funnel plot also indicated no asymmetry in the ten studies (Supplementary Fig. S2).

**Figure 3:**
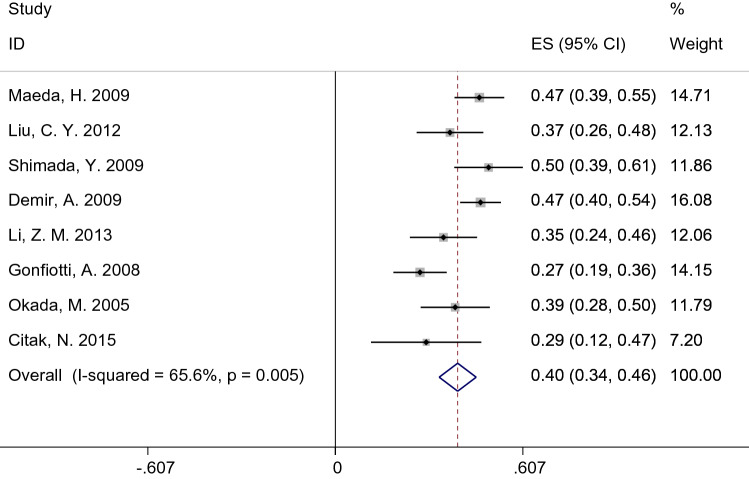
Forest plot of 5-years OS for patients diagnosed with pN1h NSCLC.

**Figure 4:**
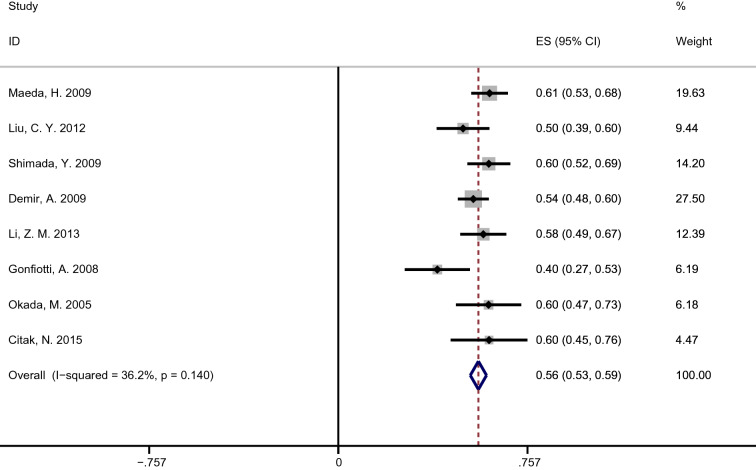
Forest plot of 5-years OS for patients diagnosed with pN1p NSCLC.

The 5-year OS of N1h and N1p groups of 8 articles were available or given, so we summarized the 5-year OS of each group, respectively. In the N1h group, the heterogeneity factor, *I*^2^ was 65.6% (*P* = 0.005), which corresponds to high heterogeneity (Fig. 3). The random-effects model yielded a pooled OS of 0.40 (95% CI 0.36–0.46). Meanwhile, we used the fixed-effects model pooled OS in the N1p group, and the pooled OS was 0.56 (95% CI 0.53–0.53; *I*^2^ = 36.2%; *P* = 0.140) (Fig. 4).

In summary, we pooled the HR of OS. The OS of N1h was significantly higher from the meta-analysis results than that of the N1p group (67%). Simultaneously, the meta-analysis's 5-year OS of N1h was 40%, and the 5-year OS of N1p was about 56%, which showed significant survival differences.

## Discussion

In this meta-analysis, we evaluated the patients in pN1 NSCLC who have different survival according to different N1 lymph node zones involvement: patients with N1p metastasis have a better prognosis than those with N1h metastasis. We found an excellent homogeneity in our ten studies, so we used the fixed-effect model. In these included studies, we found different lymph node map definitions. It did not cause significant heterogeneity. Nevertheless, we still explored the heterogeneity it might cause. The differences between lymph nodes map definition are minor, and HR as a comparison variable is hard to understand what causes the differences (Supplementary Fig. S3).

The first lymph node map, developed by Naruke during the 1960s^22,23^, was initially widely used worldwide. Subsequently, to improve the anatomical description of the Naruke map, the American Thoracic Society (ATS)^24^ map was developed. Then the Mountain-Dresler modification of the ATS map (MD-ATS)^25^ was worked out. The main difference between these two lymph node maps is that the Naruke map treats the subchondral lymph nodes along the lower border of the main bronchus as station 10(N1), and the MD-ATS map treats them as the station 7(N2). In 2009, the new international lymph node map (IASLC map) was promulgated by the IASLC^4^. Furthermore, it defined the border of #10 Hilar Nodes: “the upper border is the lower rim of the azygos vein on the right and the pulmonary artery on the left; the lower border is interlobar region bilaterally.”

The scientific study of lymphatic drainage patterns can be traced back to the early 1900s^26^. The lymph fluid produced by the lung tissue drains into the lymph nodes around the segmental bronchi and then flows into Hilar/Interlobar lymph nodes. Considering the drainage patterns of lymph nodes, the patients of the peripheral zone involved will have an earlier stage than the hilar zone involved, so it will also have better survival. IASLC presented IASLC Map and proposed lymph node stations together into “zones”, which was also proposed for future survival analyses^4^. Before the TNM of the 7th edition, IASLC analyzed the survival difference between patients with #10–11(N1p) and patients with only 12 + (N1p) (n = 522). The results showed no statistical difference, but it could be observed that the median survival time was reduced in N1h. At the same time, IASLC carried out a statistical analysis of N1h and N1p, and it was observed that the median survival time of N1p was 52 months, and that of N1h was 40 months. However, the survival difference was not statistically significant. We attempted to explore whether there was a real difference in survival between N1h and N1p patients under “the trend of statistical differences”^27^. To our knowledge, this is among the first systematic review and meta-analyses assessing the different prognoses for N1 lymph node involvement in different zones. Now, this meta-analysis revealed the different survival between N1p and N1h.

Compared with the frequently changed and improved T descriptors, there were has been little change in the N classification of lymph nodes. However, as an essential part of TNM classification, N classification is also crucial for patients’ prognosis and subsequent treatment after surgery.

In terms of the lymph node division, mang doctors are continually trying to improve and refine its descriptors (Such as statistical analysis of the single or multiple lymph nodes stations, statistics of LNR, the total number of metastatic lymph nodes). In the latest TNM Classification edition, the N1 group is divided into the N1a (single station) and N1b (multiple stations). However, we believe that only in a single station and station to distinguish between heterogeneity is still insufficient to explain the N1 patients. Based on zone N1 in installment, we put forward the N1p and N1h classification of advice.

It is well known that, to some extent, T staging is about high, and it is easy to find higher levels of N classifications. In the eighth edition of TNM classification, T1-2N1M0 was classified as IIb and T3-4N1M0 as IIIa, with a 5-year OS of 41% and 56%, respectively^3^. In this meta-analysis, we conducted a meta-summary of the individual rates of N1p and N1h. Surprisingly, the 5-year OS obtained by the meta-summary was precisely close to those of stage IIb and IIIa (40%, 56%). While this may be a statistical coincidence, it does give us a reminder as to whether the different survival of those patients between T3-4N1M0 and T1-2N1M0 is due to a higher T stage or the presence of more “N1h” patients in T3-4N1M0.

Our study's main limitation is that this meta-analysis is based on a medium sample of retrospective cohort studies. For N1 lymph node groups, especially the peripheral zone, it is not easy to assess the lymph nodes in the peripheral zone without prospective studies. One previous study reported that the median number of intrapulmonary lymph nodes (peripheral zone) retrieved increased from 2 to 5 after a novel pathology gross dissection protocol^28^. In the ten articles included in the analysis, none of the total N1 lymph nodes tested was reported, which may cause the lack of relevant information. Due to the different publication times of the articles, the included articles used different lymph node maps. Because of controversy in the definition of #10 Hilar Nodes, in the Naruke map, some lymph nodes should now be included as N2 (#7 station) are underestimated as hilar lymph nodes resulting in differences in survival. However, this difference is not reflected in the IASLC map group^4^. In the retrospective study, perhaps to explore the influence of various factors on survival, few articles independently explored the survival difference between N1p and N1h, which led to difficulties in gathering information and a lack of baseline information. This meta-analysis underlines a need for more extensive and well-conducted prospective studies evaluating patients’ survival in the pN1 stage. Future prospective studies should re-dissect the lymph nodes after lobectomy and determine a standardized anatomical protocol. Plenty of studies are needed to explore adjuvant therapy’s benefits in different N1 zones and formulate guidelines for different zones of N1 lymph nodes.

From the perspective of lymph node drainage, because the survival of the peripheral zone is better than that of the hilar zone, it can be verified laterally that tumors in the peripheral areas can be gradually transferred from the peripheral zone to the hilar zone when lymph node metastasis occurred. When performing intentional wedge resection, full consideration should be given. The anatomical segmental resection can remove the lymph nodes in the peripheral zone to determine the lymph node staging's accuracy. It is maybe a better choice than the wedge resection.

## Supplementary Information

Supplementary Information.

Supplementary Figure S1.

Supplementary Figure S2.

Supplementary Figure S3.

## Data Availability

All data generated or analyzed during this study are included in this published article (and its Supplementary Information files).

## References

[1] Duma N, Santana-Davila R, Molina JR (2019). Non-small cell lung cancer: Epidemiology, screening, diagnosis, and treatment. Mayo Clin. Proc..

[2] Goldstraw P, Crowley JJ (2006). The international association for the study of lung cancer international staging project on lung cancer. J. Thorac. Oncol..

[3] Goldstraw P (2016). The IASLC lung cancer staging project: Proposals for revision of the TNM stage groupings in the forthcoming (eighth) edition of the TNM classification for lung cancer. J. Thorac. Oncol. Off. Publ. Int. Assoc. Study Lung Cancer.

[4] Rusch VW (2009). The IASLC lung cancer staging project: A proposal for a new international lymph node map in the forthcoming seventh edition of the TNM classification for lung cancer. J. Thorac. Oncol. Off. Publ. Int. Assoc. Study Lung Cancer.

[5] Wang X (2017). Impact of omission of intrapulmonary lymph node retrieval on outcome evaluation of lung cancer patients without lymph node metastasis: A propensity score matching analysis. Clin. Lung Cancer.

[6] Liu CY, Hung JJ, Wang BY, Hsu WH, Wu YC (2013). Prognostic factors in resected pathological N1-stage II nonsmall cell lung cancer. Eur. Respir. J..

[7] Mordant P (2015). Prognostic factors after surgical resection of N1 non-small cell lung cancer. Eur. J. Surg. Oncol. J. Eur. Soc. Surg. Oncol. Br. Assoc. Surg. Oncol..

[8] Park S, Cho S, Yum SW, Kim K, Jheon S (2015). Comprehensive analysis of metastatic N1 lymph nodes in completely resected non-small-cell lung cancer. Interact. Cardiovasc. Thorac. Surg..

[9] PRISMA. http://www.prisma-statement.org/. Accessed September 16, 2020.

[10] Projects, t. H. P. o. M. P. https://hiru.mcmaster.ca/hiru/HIRU_Hedges_home.aspx. Accessed September 20, 2020.

[11] Tierney JF, Stewart LA, Ghersi D, Burdett S, Sydes MR (2007). Practical methods for incorporating summary time-to-event data into meta-analysis. Trials.

[12] Stang A (2010). Critical evaluation of the Newcastle-Ottawa scale for the assessment of the quality of nonrandomized studies in meta-analyses. Eur. J. Epidemiol..

[13] Maeda H (2009). Impact of main bronchial lymph node involvement in pathological T1–2N1M0 non-small-cell lung cancer: Multi-institutional survey by the Japan National Hospital Study Group for Lung Cancer. Gen. Thorac. Cardiovasc. Surg..

[14] Moon Y, Sung SW, Park JK, Lee KY, Ahn S (2019). Prognostic factors of pathological N1 non-small cell lung cancer after curative resection without adjuvant chemotherapy. World J. Surg..

[15] Shimada Y (2009). The prognostic impact of main bronchial lymph node involvement in non-small cell lung carcinoma: Suggestions for a modification of the staging system. Ann. Thorac. Surg..

[16] Demir A (2009). Prognostic significance of surgical-pathologic N1 lymph node involvement in non-small cell lung cancer. Ann. Thorac. Surg..

[17] Li ZM (2013). Prognostic significance of the extent of lymph node involvement in stage II-N1 non-small cell lung cancer. Chest.

[18] Gonfiotti A, Crocetti E, Lopes Pegna A, Paci E, Janni A (2008). Prognostic variability in completely resected pN1 non-small-cell lung cancer. Asian Cardiovasc. Thorac. Ann..

[19] Okada M (2005). Border between N1 and N2 stations in lung carcinoma: Lessons from lymph node metastatic patterns of lower lobe tumors. J. Thorac. Cardiovasc. Surg..

[20] Haney JC (2014). Differential prognostic significance of extralobar and intralobar nodal metastases in patients with surgically resected stage II non-small cell lung cancer. J. Thorac. Cardiovasc. Surg..

[21] Citak N (2015). The prognostic significance of metastasis to lymph nodes in aortopulmonary zone (stations 5 and 6) in completely resected left upper lobe tumors. Thorac. Cardiovasc. Surg..

[22] Naruke T, Suemasu K, Ishikawa S (1978). Lymph node mapping and curability at various levels of metastasis in resected lung cancer. J. Thorac. Cardiovasc. Surg..

[23] Naruke T (1967). The spread of lung cancer and its relevance to surgery. Nippon Kyobu Geka Gakkai Zasshi.

[24] American Thoracic Society (1983). Medical section of the American Lung Association. Clinical staging of primary lung cancer. Am. Rev. Respir. Dis..

[25] Mountain CF, Dresler CM (1997). Regional lymph node classification for lung cancer staging. Chest.

[26] Rouvière H (1929). Les vaisseaux lymphatiques des poumons et les ganglions viscéraux intrathoraciques. Ann. Anat. Pathol..

[27] Rusch VW (2007). The IASLC Lung Cancer Staging Project: proposals for the revision of the N descriptors in the forthcoming seventh edition of the TNM classification for lung cancer. J. Thorac. Oncol. Off. Publ. Int. Assoc. Study Lung Cancer.

[28] Ramirez RA (2012). Incomplete intrapulmonary lymph node retrieval after routine pathologic examination of resected lung cancer. J. Clin. Oncol. Off. J. Am. Soc. Clin. Oncol..

