# Supplementary material for: Prognostic value of different N1 lymph node zones in pN1M0 non-small cell lung cancer: a systematic review and meta-analysis
Source: Sci Rep. 2021 Nov 3;11:21606. doi: 10.1038/s41598-021-01136-2 (PMC8566486; doi:10.1038/s41598-021-01136-2)
Supplement: Supplementary file 1 — Supplementary Information. [file 41598_2021_1136_MOESM1_ESM.docx]

**Supplementary Materials**

Queries use for systematic search

KEY WORDS：NSCLC; lymph node; N1

| **Sources** | **Queries** | **Results** |
| --- | --- | --- |
| PubMed | (((((((((((Carcinoma, Non-Small-Cell Lung[MeSH Terms]) OR Carcinoma, Non Small Cell Lung[Title/Abstract]) OR Carcinomas, Non-Small-Cell Lung[Title/Abstract]) OR Lung Carcinoma, Non-Small-Cell[Title/Abstract]) OR Lung Carcinomas, Non-Small-Cell[Title/Abstract]) OR Non-Small-Cell Lung Carcinomas[Title/Abstract]) OR Nonsmall Cell Lung Cancer[Title/Abstract]) OR Non-Small-Cell Lung Carcinoma[Title/Abstract]) OR Non Small Cell Lung Carcinoma[Title/Abstract]) OR Carcinoma, Non-Small Cell Lung[Title/Abstract]) OR Non-Small Cell Lung Cancer[Title/Abstract]) AND ((N1[Title/Abstract]) OR n1[Title/Abstract])) AND ((((Lymph Nodes[MeSH Terms]) OR Lymph Node[Title/Abstract]) OR Node, Lymph[Title/Abstract]) OR Nodes, Lymph[Title/Abstract])) | 453 |
| Embase | ('lung non-small cell carcinoma cell line'/exp OR 'carcinoma, non small cell lung':ab,ti OR 'carcinomas, non-small-cell lung':ab,ti OR 'lung carcinoma, non-small-cell':ab,ti OR 'lung carcinomas, non-small-cell':ab,ti OR 'non-small-cell lung carcinomas':ab,ti OR 'nonsmall cell lung cancer':ab,ti OR 'non-small-cell lung carcinoma':ab,ti OR 'non small cell lung carcinoma':ab,ti OR 'carcinoma, non-small cell lung':ab,ti OR 'non-small cell lung cancer':ab,ti) AND ('lymph node'/exp OR 'lymph nodes':ab,ti OR 'node, lymph':ab,ti OR 'nodes, lymph':ab,ti) AND 'n1':ab,ti | 803 |
| Web of science | TS=(Carcinoma, Non-Small-Cell Lung OR Carcinoma, Non Small Cell Lung OR Carcinomas, Non-Small-Cell Lung OR Lung Carcinoma, Non-Small-Cell OR Lung Carcinomas, Non-Small-Cell OR Non-Small-Cell Lung Carcinomas OR Nonsmall Cell Lung Cancer OR Non-Small-Cell Lung Carcinoma OR Non Small Cell Lung Carcinoma OR Carcinoma, Non-Small Cell Lung OR Non-Small Cell Lung Cancer) AND TS=(Lymph Nodes OR Lymph Node OR Node, Lymph OR Nodes, Lymph) AND TS=(N1 OR n1) | 657 |
| Cochrane | （（MeSH descriptor: [Carcinoma, Non-Small-Cell Lung] explode all trees OR ((carcinoma, non small cell lung):ab,ti,kw OR (carcinomas, non-small-cell lung):ab,ti,kw OR (lung carcinoma, non-small-cell):ab,ti,kw OR (lung carcinomas, non-small-cell):ab,ti,kw OR (non-small-cell lung carcinomas):ab,ti,kw OR (nonsmall cell lung cancer):ab,ti,kw OR (non-small-cell lung carcinoma):ab,ti,kw OR (non small cell lung carcinoma):ab,ti,kw OR (carcinoma, non-small cell lung):ab,ti OR (non-small cell lung cancer):ab,ti,kw) AND (MeSH descriptor: [Lymph Nodes] explode all trees OR ((Lymph Node):ab,ti,kw OR (Node, Lymph):ab,ti,kw OR (Nodes, Lymphl):ab,ti,kw)) AND ((N1):ab,ti,kw) | 33 |
