# Supplementary material for: Prognostic value of different N1 lymph node zones in pN1M0 non-small cell lung cancer: a systematic review and meta-analysis
Source: Sci Rep. 2021 Nov 3;11:21606. doi: 10.1038/s41598-021-01136-2 (PMC8566486; doi:10.1038/s41598-021-01136-2)
Supplement: Supplementary file 3 — Supplementary Figure S2. [file 41598_2021_1136_MOESM3_ESM.pdf]

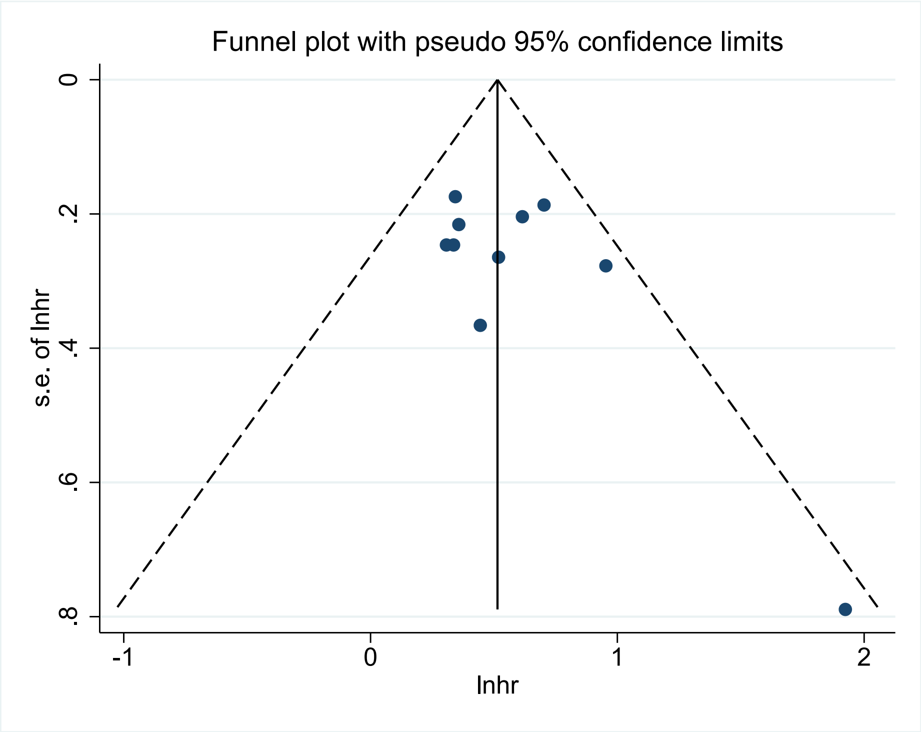

Supplementary Figure 2. Funnel plot for the analysis of publication bias between overall survival of patients diagnosed with pN1 NSCLC and involved with N1h versus N1p
